# Bee Venom and Its Component Apamin as Neuroprotective Agents in a Parkinson Disease Mouse Model

**DOI:** 10.1371/journal.pone.0061700

**Published:** 2013-04-18

**Authors:** Daniel Alvarez-Fischer, Carmen Noelker, Franca Vulinović, Anne Grünewald, Caroline Chevarin, Christine Klein, Wolfgang H. Oertel, Etienne C. Hirsch, Patrick P. Michel, Andreas Hartmann

**Affiliations:** 1 Université Pierre et Marie Curie-Paris 6, UMR_S 975 - UMR 7725, Centre de Recherche en Neurosciences, ICM, Therapeutique Experimentale de la Neurodegenerescence, Paris, France; 2 Inserm, U 975, Centre de Recherche en Neurosciences, ICM, Therapeutique Experimentale de la Neurodegenerescence, Paris, France; 3 CNRS, UMR 7225, Centre de Recherche en Neurosciences, ICM, Therapeutique Experimentale de la Neurodegenerescence, Paris, France; 4 ICM, Therapeutique Experimentale de la Neurodegenerescence, Paris, France; 5 Department of Neurology, Philipps-University Marburg, Marburg, Germany; 6 Unité Mixte de Recherche S677, Université Pierre et Marie Curie-Paris 6, Paris, France; 7 Département de Neurologie, Pôle des Maladies du Système Nerveux, Hôpital de la Pitié-Salpêtrière, Paris, France; 8 Institute of Neurogenetics, University of Lübeck, Lübeck, Germany; 9 Department of Psychiatry, University of Lübeck, Lübeck, Germany; National Institutes of Health, United States of America

## Abstract

Bee venom has recently been suggested to possess beneficial effects in the treatment of Parkinson disease (PD). For instance, it has been observed that bilateral acupoint stimulation of lower hind limbs with bee venom was protective in the acute 1-methyl-4-phenyl-1,2,3,6-tetrahydropyridine (MPTP) mouse model of PD. In particular, a specific component of bee venom, apamin, has previously been shown to have protective effects on dopaminergic neurons *in vitro*. However, no information regarding a potential protective action of apamin in animal models of PD is available to date. The specific goals of the present study were to (i) establish that the protective effect of bee venom for dopaminergic neurons is not restricted to acupoint stimulation, but can also be observed using a more conventional mode of administration and to (ii) demonstrate that apamin can mimic the protective effects of a bee venom treatment on dopaminergic neurons. Using the chronic mouse model of MPTP/probenecid, we show that bee venom provides sustained protection in an animal model that mimics the chronic degenerative process of PD. Apamin, however, reproduced these protective effects only partially, suggesting that other components of bee venom enhance the protective action of the peptide.

## Introduction

Parkinson disease (PD) is a neurodegenerative disorder characterized by typical motor symptoms (akinesia, rigidity, rest tremor) that result from the progressive loss of dopamine (DA) neurons in the substantia nigra (SN) [1]. Although symptomatic therapy can provide benefit for many years, the disorder progresses slowly, eventually resulting in significant disability [2]. Strategies to delay onset or slow progression of PD are therefore needed to provide long-term therapeutic benefit to PD patients.

Recent studies suggest that bee venom (*apis mellifera*) can protect dopaminergic (DA) neurons from degeneration in experimental PD. In particular, it has been observed that bilateral acupoint stimulation of lower hind limbs with bee venom was protective in the acute 1-methyl-4-phenyl-1,2,3,6-tetrahydropyridine (MPTP) mouse model of PD [3] [4]. This observation is of interest in view of possible pharmacological interventions aimed at modifying disease progression. Yet, the molecular target of this effect requires to be characterized since bee venom is composed of many chemical agents including polypeptides, enzymes and amines [5]. Furthermore, it remains to be established whether the protective effect of bee venom is specific to administration through acupoint stimulation.

Interestingly, two studies reported that the peptide apamin, a specific component of bee venom, can protect DA neurons in a model system of midbrain cultures that mimics the selective demise of these neurons in PD [6] [7]. The protective effect of apamin was attributed to a small increase in excitability of the DA neurons that caused a moderate and persistent elevation in cytosolic calcium [6]. This is consistent with the known pharmacological properties of apamin as a potent and irreversible blocker of Ca^2+^-activated K+ (SK) channels. These channels link intracellular calcium transients to changes of the membrane potential by promoting K^+^ efflux following increases of intracellular calcium during an action potential [8]. This observation fits with the more general idea that electrically active DA neurons might be also more resilient to toxic insults in a degenerative context [9] [10]. Most importantly, this indicates that apamin is possibly a crucial component in bee venom regarding its protective action on DA neurons *in vivo*. Still consistent with this possibility, apamin can penetrate the blood-brain barrier when injected peripherally despite its peptide structure [11]. Until now, there is, however, no information regarding a potential protective action of apamin in animal models of PD.

The specific goals of the present investigation were the following; (i) To establish that the protective effect of bee venom on DA neurons is not restricted to acupoint stimulation and can also be observed using a more conventional mode of administration, i.e., intra-peritoneal injections. (ii) To demonstrate that apamin can mimic to some extent the protective effects of a bee venom treatment on DA neurons. (iii) To refine and complete our understanding of the underlying mechanism(s), particularly in view of possible anti-inflammatory effects associated with bee venom [4] and apamin [12] treatments. (iv) To determine whether neuroprotective treatments result in improved motor performances in MPTP-lesioned animals. To carry out these experimental protocols, we used a chronic mouse model of MPTP intoxication which closely mimics the progressive loss of DA neurons in PD as previously described [13] [14].

## Materials and Methods

### Animals

Animals were housed, handled, and cared for in accordance with the Guide for the Care and Use of Laboratory Animals [NCR (National research council) 1996] and the European Union Council Directive 86/609/EEC, and the experimental protocols were approved by the Comité régional d'éthique pour l'expérimentation animale, Ile-De-France – Paris – Comité 3 (Dossier p3/2008/057), the Regierungspräsidium Gießen (82/2009), and the Ministerium für Energiewende, Landwirstschaft, Umwelt und ländliche Räume des Landes Schleswig-Holstein (V312-7224.122-20 99-7/12). For all studies, mice were maintained on a 12∶12 h light/dark cycle with lights on at 6.30 a.m. The room temperature was kept at 23°C, with free access to standard diet and tap water.

### Chronic MPTP intoxication of mice

**Figure 1:**
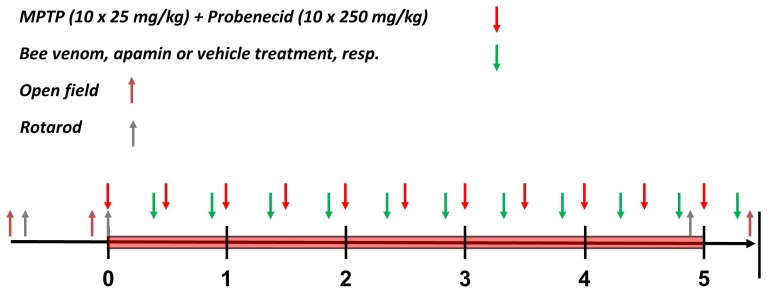
Time schedule for bee venom and apamin injections in MPTP/probenecid-intoxicated mice. Time points at which MPTP/probenecid (red arrows) and bee venom or apamin (green arrows) injections were carried out. Note that bee venom and apamin were given every 3.5 days, starting 48 h day after the first MPTP/probenecid injection. Blue and grey arrows depict time points at which mice were taken for the open field or rotarod testing, respectively. Habituation to the open field test and training to the rotarod test were carried out 3 and 2 days before beginning the first MPTP/probenecid injection, respectively. Baseline values for the open field and rotarod tests were acquired one day before, and on the same day as the first injection with MPTP/probenecid, respectively. For the rotarod testing, post-treatment values were obtained one day after the ninth injection with bee venom or apamin and for the open field testing 120 min after the last injection with of one of these two treatments. After the open field testing, all mice were killed for brain processing and analysis.

For this study, we used the MPTP/probenecid paradigm of intoxication which represents one of the most stable and chronic toxin-based mouse models of PD available today [14] [15]. We used 9-week old male C57/Bl6 mice (Janvier Breeding Center, Le Genest St Isles, France) which received 10 repeated intraperitoneal (i.p.) injections of MPTP/probenecid or vehicle carried out every 3.5 days over a time period of 5 weeks. Mice received i.p. injections of bee venom or saline (vehicle) 48 h after the first MPTP/probenecid injection using a time interval of 3.5 days between two consecutive injections. During MPTP intoxications mice were kept in a warming, ventilated cabinet with temperature set to 32°C to maintain body temperature. Mice (n = 8–10 treatment group) were sacrificed 24 h after the last injection of bee venom or apamin (Fig. 1).

### Treatment

Amounts of bee venom and apamin given to mice were chosen by extrapolating from dosages used in human desensitisation protocols. Two series of experiments were conducted. Two different dosages of bee venom (Alyostal) (low = 12 µg/kg/BW; high = 120 µg/kg/BW) and apamin (low: 0.5 µg/kg/BW; high 1.0 µg/kg/BW) were used for the assessment of brain lesions in the first set of experiments where we analysed neurotransmitters and cell numbers. In the second set of experiments used for behavioural analysis, only high dosages of bee venom (120 µg/kg/BW) or apamin (1.0 mg/kg/BW) were given based on the results of the first series. The second group of mice was also used for the assessment of striatal cytokines by ELISA. A subset of animals received only one injection of MPTP and high dosage of bee venom, high dosage of apamin or saline, respectively, for analysis of striatal complex I activity. These animals were killed 120 min after injection of compounds and brains were processed for further analysis. The same protocol was applied for striatal MPP^+^ measurements, where survival times were 90 and 150 minutes, respectively, after a single injection of MPTP/probenecid, preceded by bee venom, apamin or saline injections 36 h before. Mortality ranged between 7.3% in the first set and 23% in the second set of experiments without a clear explanation for higher mortality in the second set of experiments.

### Neurochemical analysis

Neurochemical analysis was performed as previously described [14]. For catecholamine (DA, DOPAC, HVA) quantification, mice were killed and brains were rapidly removed. The striatum was dissected and placed into 250 µL 0.1 N perchloric acid containing 0.05% disodium EDTA and 0.05% sodium metabisulfite. Brain tissue pieces were then sonicated for 10 s and the resulting homogenates were centrifuged at 13,000 g for 20 min at 4°C. Supernatants were filtered through a 0.2 µm membrane and filtrates were stored at −80°C until further analysis. Before injection into high-performance liquid chromatography (HPLC), 100 µl of the samples were mixed with 10 µL phosphate buffer (2 M, pH 7.5) and 5 µL ascorbic acid (0.3 mg/mL). The potential for electrochemical detection was set at +0.65V and the column temperature was maintained at 19°C. The mobile phase (a buffered aqueous solution containing 15.9% methanol, 1.25 mM octane-1 sulfonic acid sodium salt and 76% of a buffer containing 0.7 M KH_2_PO_4_, 1 mM EDTA, 31 mM triethylamine, at pH 3) was delivered onto a reversed phase C18 column (250×4.6 mm, bonded silica, Sunfire, Waters, Guyancourt, France). Striatal levels of DA ranged from 6.2 to 9.7 ng/mg wet tissue in saline/saline treated mice.

MPP^+^ levels were detected as previously described [14], for further details see Supporting Information S1.

### ELISAs for IL-1β, IL-6, TNF-α in the striatum

The left striatum of animals killed one day after the last injection was used for cytokine quantification by ELISA. The striata were incubated with a solution of T-Per (Pierce, Rockford, USA) and Complete Protease Inhibitor Cocktail Tablet (Roche Diagnostics, Mannheim, Germany) and homogenized mechanically. After centrifugation, the supernatant was collected. Concentrations of cytokines (IL-1β, IL-6, TNF-α) were measured by using the Duo Set ELISA Development Systems Mouse IL-1β/IL-1F2, Mouse IL-6 and Mouse TNF-α (R&D Systems, Minneapolis, USA). ELISAs were used according to the manufacturer's protocol except that SuperBlock Blocking Buffer in TBS (Pierce Biotechnology, Rockford, USA) was used for blocking the IL-1β ELISA.

### Immunohistochemistry

The posterior part of the brains were postfixed (4% PFA), and cryoprotected. Immunohistochemistry was performed as previously described [16] on free-floating cryomicrotome-cut sections (20 µm). Sections were incubated with a polyclonal antibody against TH (1∶1000; Pel-Freez Biologicals, Rogers, AR) and a secondary antibody (Vectastain, Vector Laboratories, Burlingame, CA). Absolute numbers of tyrosine hydroxylase-positive (TH+) neurons in the SN pars compacta (SNpc) were determined unbiased by cell size and by the volume of the structures with an automatic stereology system (CAST Grid, Olympus Denmark, Albertslund, Denmark) on a Leica DM RB microscope (Leica Microsystems, Bensheim, Germany) using an optical fractionator as previously described [16]. In brief, cell counts were quantified stereologically on regularly spaced sections covering the whole rostrocaudal extent of the SN with the VisioScan stereology tool and cell loss was verified by Nissl counterstaining in the high dosages group. Tissue was serially cut with a random start. Counting fraction covered 30% of the SNpc. The SN was identified according to established anatomical landmarks (corresponding to the atlas of Franklin and Paxinos, [17]). Absolute values of TH+ neurons in saline/saline treated animals ranged from 4680 to 6000 (mean 5112±203). To define the border between SN pars compacta and VTA, the medial lemniscus was taken as a landmark. Neurons were distinguished from glia using morphological criteria as previously described [18], [19]. Neurons were identified on the basis of size and a clearly visible nucleolus and surrounding cytoplasm [18].

### Behavioural analysis

Rotarod testing was performed with a system equipped with a rod diameter of 7.3 cm (rat-size) (TSE-Systems, Bad Homburg, Germany). Testing was performed at a rotation speed of 10 and 20 rpm (n = 6–10 per group). All mice were trained 48 h before the first test. Training was considered effective when either 5 consecutive runs at 10 rpm had been completed or when mice were able to maintain themselves for 240 sec on the rotating rod. Baseline values were obtained at 10 rpm under the same conditions on the day prior to the first MPTP/probenecid injection. Post-treatment values were obtained after the ninth bee venom/apamin/vehicle injection.

Motor track length was recorded using a computerized open field (80×80 cm) system (Viewer II, Biobserve, Bonn, Germany). The test started 5 min after mice had been placed into the open field boxes. Recordings were performed over a time period of 25 min. Note that mice were placed for habituation 72 h before the first test session. Baseline values were recorded one day before the first injection of MPTP/probenecid. Post-treatment values were recorded 120 min after the last injection of bee venom/apamin/vehicle and thus, 24 h before mice were killed.

### Complex I activity

Mitochondrial respiratory chain complex I (Cx I) and citrate synthase (CS) activity were measured as previously described [20], [21]. In brief, after killing the animals (n = 4 per group) striata of both hemispheres were dissected and snap frozen in liquid nitrogen. Tissue was homogenised in homogenization buffer (250 mM sucrose, 10 mM Tris, pH 7.4, 1 mM EDTA), nuclei and cell debris were removed by centrifugation at 1,500 g, and mitochondria were isolated by centrifugation at 11,800 g. The mitochondrial pellet was then resuspended in homogenization buffer. Cx I and CS activities were measured by spectrophotometric methods in these mitochondrial preparations as reported [20], [21]. Data were expressed as ratios of Cx I/CS activity. Analysis was performed in 4 independent runs, whereat for each run one randomly chosen sample of each group was analysed. In each run Cx I activity of saline/saline treated animals was set as 100% and data were expressed in percentage of saline/saline group.

### Statistical analysis

Data are expressed as the percentage of corresponding values of saline/saline treated animals that were set as 100%. Each data point represents mean ± S.E.M. Multiple comparisons against a single reference group were performed by one-way ANOVA followed by a post-hoc Dunnett's test. When all pairwise comparisons were carried out, one-way ANOVA was followed by a post-hoc Holm-Sidak test. For behavioural analysis, a two-way ANOVA for repeated measurements was used, followed by a post-hoc Holm-Sidak test for pairwise comparisons. The null hypothesis was rejected at an α risk of 5%. A table with all degrees of freedom, F and p values is given in the supplemental materials (Table S1).

## Results

### Bee venom and apamin protect against MPTP-induced DA cell loss

**Figure 2:**
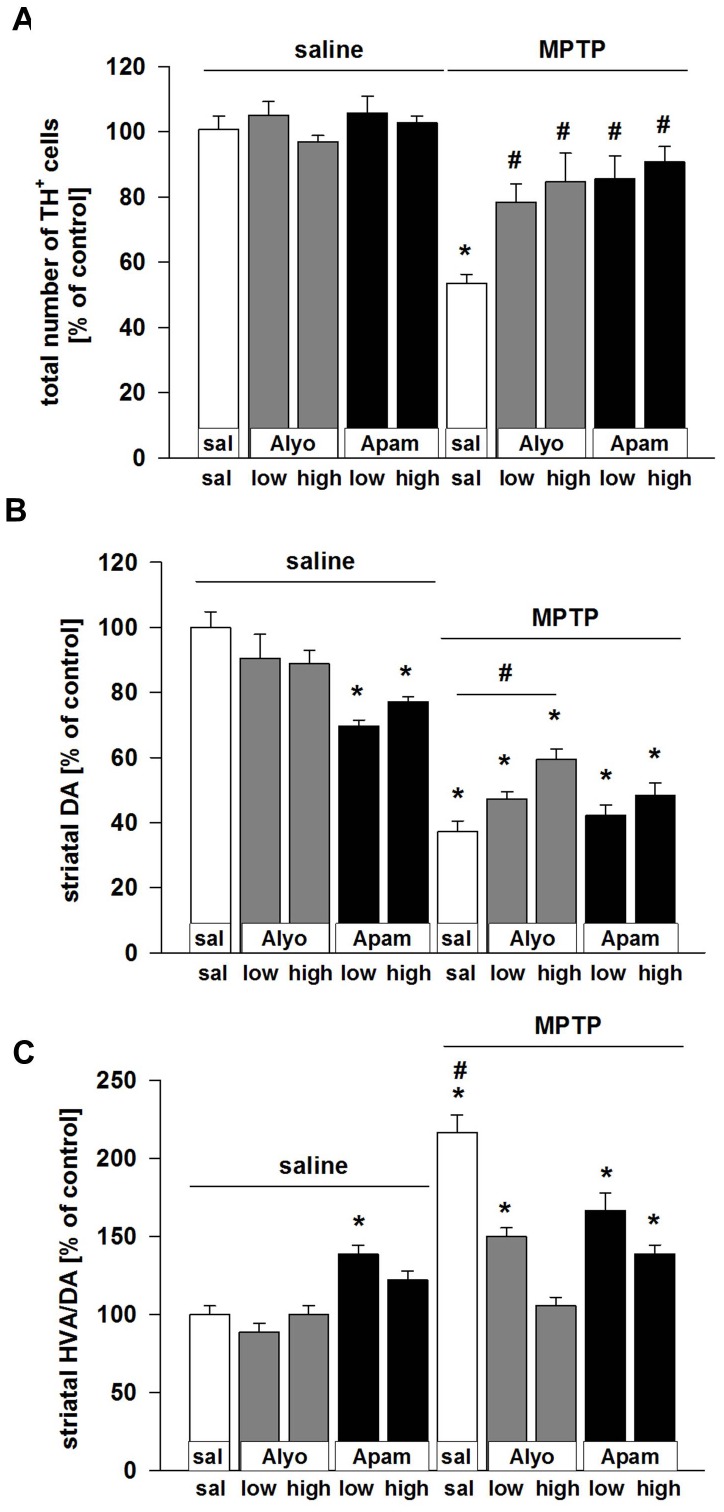
Effect of bee venom (Alyo) and apamin (Apam) treatments against MPTP intoxication. (A) Number of SNpc TH+ cells of the right hemisphere in MPTP/probenecid-treated mice receiving or not treatments with bee venom or apamin. Legends corresponding to various treatment paradigms are given above. (B) Striatal DA levels and (C) HVA/DA ratios in mice receiving the same treatment regimens. Data are expressed as percentage of saline-injected mice. Actual values for controls were as follows: TH^+^ cell numbers 5112.5±203.3, striatal DA levels, 8.82 ng/mg wet tissue ±0.42; HVA/DA ratios, 0.18±0.01. Data represent mean values ± s.e.m of 5–8 animals. *p<0.05 compared to control values, #p<0.05 for pairwise comparison.

TH cell counts in the SNpc showed that both bee venom and apamin protected nigral DA neurons from MPTP/probenecid intoxication. This effect was significant at both dosages of either compound (Fig. 2a). Also, Nissl counterstains revealed that loss of TH+ neurons in the SNpc corresponded to actual cell loss and not only to a transient down regulation of TH expression (MPTP/saline-treated animals 98.8%±8.7%, MPTP/apamin-treated animals 95.3%±9.2%, and MPTP/alyostal-treated animals 96.8%±8.2% compared to saline/saline treated animals in which the total number of TH negative neurons ranged from 1884 to 2188 cells, mean 2086±78,7, Fig. S3).

### Bee venom and apamin increase striatal dopamine levels after MPTP intoxication

Striatal DA levels determined by HPLC reflected protection of DA nerve terminals. Observed effects appeared dose-dependent but significance was only reached in the bee venom group receiving high dosages (Fig. 2b). Surprisingly, apamin but not bee venom caused a decrease in striatal DA concentrations in control animals (Fig. 2b). Striatal HVA/DA ratios which provide an index of DA turnover in dopaminergic nerve terminals were increased in MPTP/probenecid-treated mice and lowered by either apamin or bee venom. HVA/DA levels were, however, increased by apamin (but not by bee venom) in control animals (Fig. 2c). Similar results were obtained in striatal DOPAC/DA ratios. Whereas MPTP/probenecid treatment increased values, both apamin and bee venom significantly reduced values to control levels (Fig. S2). To exclude that treatment with either bee venom or apamin alter striatal MPP+ levels, these were analysed after pre-treatment with either compound 36h before MPTP/probenecid injection according to the study design. As expected, they remained unaltered by the compounds (Fig. S1).

### Bee venom and apamin exert paradoxical behavioural effects after MPTP intoxication

**Figure 3:**
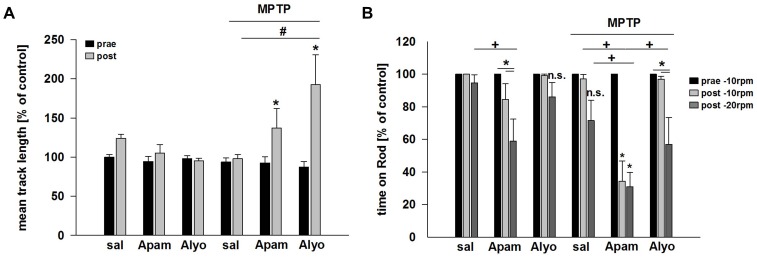
Behavioural effects of bee venom (Alyo) and apamin (Apam) in mice exposed or not to repeated MPTP/probenecid injections. (A) Track length reached in the open field after 25 min (100% = 6998 cm±226) and (B) rotarod performance (100% = 240 seconds) as a function of the treatments mentioned above. Baseline values (prae) were obtained at 10 rotations per minute (rpm). Post-treatment values were obtained both at 10 and 20 rpm after the ninth bee venom/apamin/vehicle injection (Fig. 1). Data are expressed as mean ± s.e.m. *p<0.05 compared to control values, #p<0.05 for pairwise comparison.

Behavioural testing was also carried out in apamin-treated, bee venom-treated mice, and control groups prior to the repeated MPTP/probenecid injections and at the end of the treatment regimen, 24 h before killing the animals. Neither bee venom nor apamin produced a significant effect in mice assessed with the open field test which measures exploratory behaviour and general activity in rodents [22]. MPTP did not slow the animals significantly compared to control animals but mice treated with MPTP/apamin or MPTP/bee venom were more active than MPTP/saline-treated animals (Fig. 3a). In the rotarod test which measures motor planning and movement coordination, we could not observe deficits in mice treated with MPTP/probenecid, only. However, mice treated with MPTP/probenecid and receiving apamin spent significantly less time on the spindle than MPTP/saline-treated animals (Fig. 3b).

### Bee venom but not apamin reverse changes in pro-inflammatory cytokine production after MPTP intoxication

**Figure 4:**
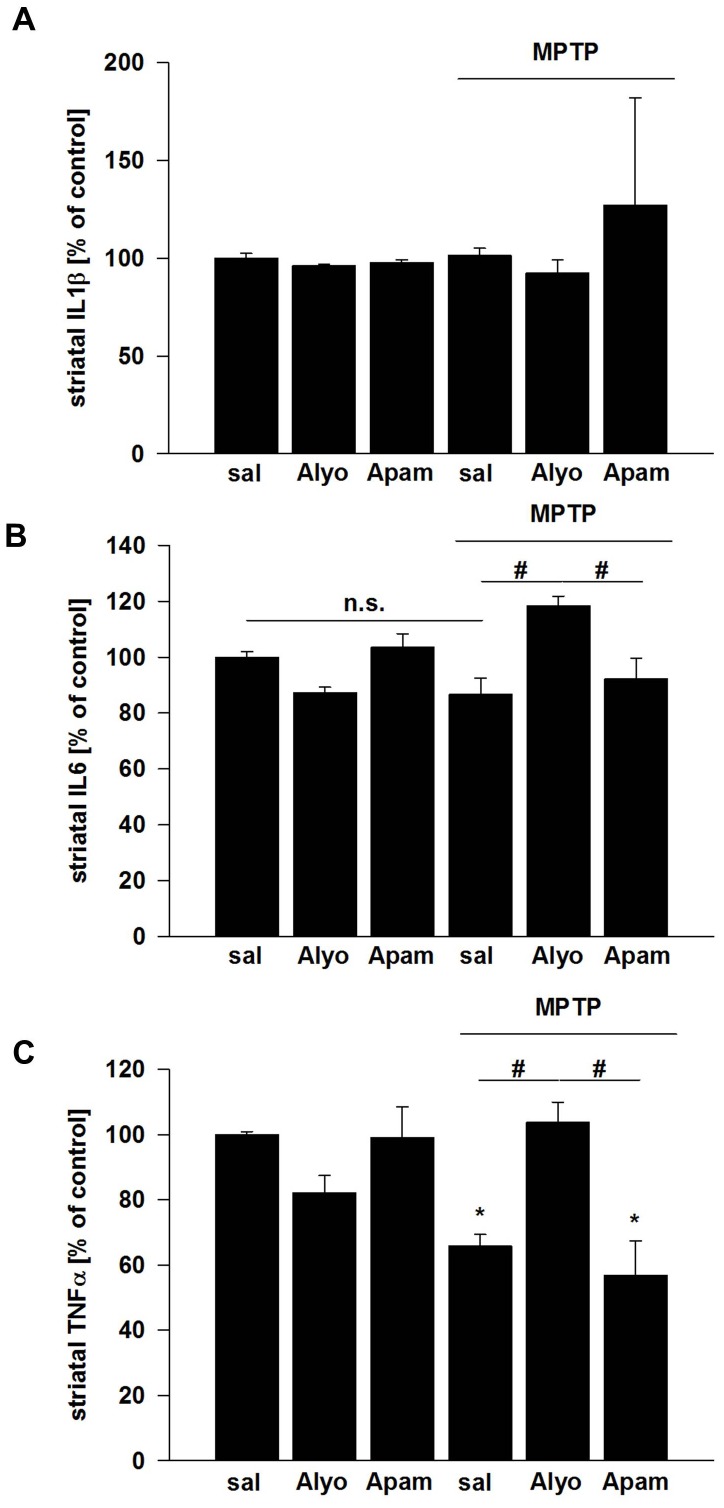
Effects of bee venom (Alyo) and apamin (Apam) on striatal cytokine levels in mice exposed to repeated MPTP/probenecid vs. saline injections. (A) IL-1β, (B) IL-6 and (C) TNF-α striatal levels. Data are expressed as mean ± s.e.m. *p<0.05 compared to control values, #p<0.05 for pairwise comparison.

Cytokine ELISAs of striatal extracts showed no differences for IL-1β and IL-6 neither after MPTP/probenecid intoxication nor after treatment with apamin or bee venom (Fig. 4 a,b). However, striatal TNF-α levels were reduced after MPTP/probenecid intoxications. Whereas apamin treatment did not influence this alteration in TNF-α levels, bee venom reversed this effect (Fig. 4c).

### Bee venom and apamin do not alter Cx I activity

**Figure 5:**
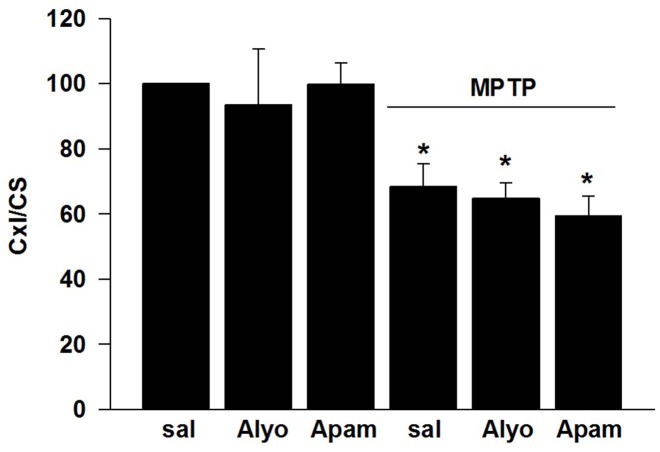
Effects of bee venom (Alyo) and apamin (Apam) on striatal Cx I activity in mice exposed to repeated MPTP/probenecid vs. saline injections. The ratio of Cx I/CS activity is given in the graph as percentage of saline/saline animals with n = 4 per group. Analysis was performed in four independent experiments whereas in each experiment the obtained value of the saline/saline group (expressed as nmol/ml*min) was set to 100%. Data are expressed as mean ± s.e.m. *p<0.05 compared to control values, #p<0.05 for pairwise comparison.

While MPTP treatment significantly reduced striatal Cx I activity and by that showing reliability of the test, neither apamin nor bee venom influenced its activity under control conditions (saline treatment) nor after MPTP/probenecid intoxication (Fig. 5).

## Discussion

Here, we show that high dosages of bee venom administered through the i.p. route provide substantial protection to SNpc DA neurons and striatal terminals in a chronic mouse model of PD. High dosages of the bee venom peptide apamin afforded the same level of protection to DA cell bodies. Apamin failed, however, to significantly preserve DA nerve terminals even if a tendency for protection was also observed which is mirrored by the significantly reduced quotient of HVA/DA as well as DOPAC/DA. Of importance, protective effects of bee venom and apamin appeared not related to a possible anti-inflammatory action of these treatments. Whereas MPTP decreased Cx I activity as expected, neither apamin nor bee venom altered its activity, suggesting that neuroprotection was not due to a direct impact on respiratory chain function.

### Protection of DA neurons by bee venom is not restricted to acupoint stimulation

Recent studies suggest that bee venom can protect DA neurons from degeneration in experimental PD. In particular, it has been observed that bilateral acupoint stimulation of lower hind limbs with bee venom was protective in the acute MPTP mouse model of PD [4] [3]. Our present data clearly indicate that the protective effect of bee venom for DA neurons is not restricted to this mode of administration as neuroprotection was also detected using the more conventional i.p. route. Most importantly, this effect was observed in the MPTP/probenecid paradigm of intoxication, which represents a chronic, progressive toxin-based mouse model of PD with substantial loss of DA neurons [14] [15]. In contrast to what occurs in the acute paradigms of MPTP intoxication, DA neurons do not recover from repeated injections of MPTP/probenecid [13], which suggests that bee venom had primarily a neuroprotective effect and did not simply restore TH expression in a population of diseased neurons.

### Bee venom-mediated protection is partially reproduced by apamin

Apamin can protect DA neurons in a model system of midbrain cultures that mimics the selective demise of these neurons in PD [6] [7]. This observation raised the possibility that the protective effects of bee venom for DA neurons in MPTP/probenecid-treated mice might result from the presence of this peptide in the venom. Accordingly, apamin was found as effective as bee venom in rescuing DA cell bodies in MPTP/probenecid-intoxicated mice. Yet, the peptide did not provide significant protection to dopaminergic nerve endings suggesting that another molecule in bee venom might enhance the action of apamin, especially at the axonal level.

Mellitin, a small peptide which represents 40 to 60% of dry whole honeybee venom, is a candidate molecule potentially able to enhance the neuroprotective action of apamin in mice receiving bee venom. Mellitin has several interesting pharmacological properties. In particular, as a result of its interaction with negatively changed phospholipids, mellitin inhibits the activity of the Na/K-ATPase [23] [24]. This effect might be relevant in the context of this study as partial inhibition of the enzyme pump was reported to provide a partial rescue to DA neurons in culture [6]. Apart from this action, melittin was reported to restore the activity of the ubiquitin-proteasome system in an animal model of ALS [25]. Thus, preservation of the proteasomal function by melittin could also potentially improve the protection provided by apamin. This is particularly relevant in the context of DA cell death as this enzymatic complex is thought to be dysfunctional in PD [26]. It is not known, however, whether melittin can cross the blood-brain barrier [27] with an efficacy comparable to that of apamin [11].

It must be pointed out that the cumulative amounts of apamin administered through the high dosage regimen were not equivalent to the presumed amounts of apamin in the high dosage regimen for bee venom. This suggested that higher dosages of apamin could possibly have further improved neuroprotection of DA neurons. The amounts of apamin used in the present study, however, were justified by the observation that MPTP/probenecid-treated mice receiving the highest dosage of the peptide were trembling. Thus, the therapeutic range of pure apamin and apamin contained in bee venom seemed to differ notably.

### Mechanisms underlying neuroprotection by bee venom and apamin in the chronic model of MPTP intoxication

Apamin exerted its rescuing effect most likely via a direct action on DA neurons since two SK channel subtypes, namely SK2 and SK3 channels which are predominantly blocked by apamin [28], are present in these neurons [29] [30]. Based on results obtained in midbrain cultures, it was proposed that blockade of SK channels on DA neurons leads to the activation of a calcium-dependent signalling pathway required to prevent apoptosis [6]. Accordingly, blockade of voltage-gated T-type calcium channels by flunarizine abolished the effect of apamin [6]. An anti-apoptotic effect of bee venom was also reported in SH-SY5Y human neuroblastoma cells, which suggests that one or several components of bee venom acted directly on neuronal cells to prevent their demise [31]. Protection might be partly due to apamin in this preparation but the presence of SK channels has not been established in SH-SY5Y cells. The possible participation of other bee venom components in neuroprotection has not been addressed yet.

Despite the arguments in favour of direct effects of bee venom and apamin on DA neurons, the possibility of an indirect action of these treatments needs to be considered. Indeed, it was reported that bee venom [32] [4] and apamin [12] have the potential to repress inflammatory processes mediated by microglial cells. While the anti-inflammatory effects of apamin are unequivocally mediated by blockade of SK channels present on microglial cells [12], the possible intervention of SK channels in the bee venom effect has not been addressed yet [4].

In contrast to previous reports on changes in striatal cytokines in the MPTP/probenecid model of PD [33], chronic MPTP/probenecid intoxication in the present study was not accompanied by an increase in striatal inflammatory markers – rather the opposite, at least after 5 weeks of treatment. Whereas levels of IL-6 and IL-1β remained unchanged, levels of TNF-α were decreased after MPTP/probenecid intoxication. However, the MPTP/probenecid model of PD may not be an appropriate model to study neuroinflammation, although differing results among groups deserve explanation. Meredith et al. (2008) and Luchtman et al. (2009) report nigral microglial activation and striatal cytokine level increases (IL-1 and TNF-alpha) in the MPTP/probenecid model [33], [34], respectively, whereas Alvarez-Fischer et al. (2008) failed to detect activated microglial cells both in the SNpc and the striatum [14]. Also, the post sacrifice time point at 5 weeks may not reflect possible changes along the time course. However, predictably, cytokine levels would at least be expected to be the same as baseline in the MPTP-treated groups – however, they were even lower in the MPTP/saline and MPTP/apamin-treated groups, which is surprising. The most likely explanation, at present, is that the MPTP/probenecid model is not the most appropriate PD model to study neuroinflammation. However, MPTP/probenecid induced decreased in striatal TNF-α levels were reversed by bee venom to control level, suggesting a possible explanation for different effect size of both treatments. Note, however, that both direct and indirect effects are conceivable in the pathophysiological context of PD where ongoing inflammatory processes contribute to DA cell death [35].

According to the general idea that electrical activation of DA neurons improve their resistance to toxic insults that is at least in part dependent on alteration of cellular respiration [9], [10], we hypothesized that treatment with either compound (apamin or bee venom) could affect changes on Cx I activity induced by the Cx I inhibitor MPTP. Although we were predictably able to detect changes induced by MPTP treatment, bee venom and apamin treatment remained without any effect on the respiratory chain.

### Behavioural testing in bee venom and apamin-treated mice

DA cell loss did not result in significant impairment of motor parameters in MPTP/probenecid- treated mice. This is a known limitation of rodent MPTP models of PD, probably because DA cell loss and accompanying striatal DA depletion is insufficient to induce a behavioural phenotype. Others have reported deficits in the grid test and the open field test in MPTP/probenecid-treated mice [15] [33] but levels of striatal DA depletion achieved in these studies were far greater than those reported here. Of interest, however, both apamin and bee venom stimulated the locomotor activity in mice intoxicated with MPTP/probenecid, indicating that in this experimental situation, both treatments probably had the capacity to stimulate DA release [36], possibly by a mechanism involving SK channel blockade. Hyperactivity observed in the open field assay might also be related to an increase in adrenergic/noradrenergic release induced by. SK channels are present centrally on locus coeruleus neurons [37] and peripherally on the adrenal gland and their blockade can cause an increase in adrenergic tone [38].

Incidentally, we also observed that mice treated with MPTP/probenecid and receiving the highest dosage of apamin were trembling, an effect possibly attributable to a hypercholinergic tone in the striatum. Indeed, SK channel blockade was reported to stimulate the bursting activity of striatal cholinergic interneurons [39], an effect which most likely stimulates acetylcholine release within the striatum. Therefore, the onset of tremor in MPTP/probenecid-treated mice receiving apamin might signify that the peptide revealed a hypercholinergic state consecutive to dopaminergic denervation. This seems also the most likely explanation for poor rotarod performances of apamin-treated animals which, due to tremor, lost grip more readily than saline- or bee venom treated animals.

In summary, our data suggest that bee venom can induce sustained protection of dopaminergic neurons in an animal model that mimics the chronic degenerative process of PD. The bee venom peptide apamin, a specific blocker of SK channels, only partially reproduced these protective effects, suggesting that another bee venom component enhances the protective action of the peptide in bee venom. Moreover, it induces a decline in striatal dopamine in control animals, possibly indicating a harmful potential in non-PD affected individuals that is not present in the whole protein mixture of bee venom. Finally, note that we are currently conducting an exploratory clinical trial of bee venom in PD patients with moderately advanced disease (ClinicalTrials.gov Identifier NCT01341431) which will end by autumn of 2013.

## Supporting Information

Figure S1 **Effects of bee venom (Alyo) and apamin (Apam) on striatal MPP^+^ levels.** The figure shows striatal MPP^+^ levels at 90 and 150 minutes after MPTP/probenecid treatment and 36 hours after bee venom, apamin or saline treatment, respectively. At no time point MPP^+^ levels were altered by the treatment.
(TIF)

Figure S2 **Striatal DOPAC/DA ratios in MPTP/probenecid-treated mice treated with bee venom (Alyo), apamin (Apam) or saline (Sal).** Data are expressed as percentage of saline-injected mice. Actual values for saline/saline treated animals DOPAC/DA ratio were 0.59±0.05. Data represent mean values ± s.e.m of 5–8 animals. *p<0.05 compared to control values, #p<0.05 for pairwise comparison.
(TIF)

Figure S3 **Total number of neurons in the SNpc.** The graph represents the absolute number of TH^+^ (black) and TH^−^ (grey) neurons in the SNpc of mice treated with saline/saline, MPTP/saline, MPTP/high Alyostal, and MPTP/high Apamin. Data represent mean of 5–8 animals per group.
(TIF)

Supporting Information S1 In the supporting information section technical details of MPP+ analysis after pre-treatment with either bee venom or apamin are specified.
(DOC)

Table S1 **Statistical details.** The table gives all statistical details as dependent variable, the performed test (all ANOVAs were followed by a post-hoc Holm-Sidak test for all pairwise comparisons), the degrees of freedom (dF), F, and p-values.
(DOCX)
