# Supplementary figures and images for: Bee Venom and Its Component Apamin as Neuroprotective Agents in a Parkinson Disease Mouse Model
Source: PLoS One. 2013 Apr 18;8(4):e61700. doi: 10.1371/journal.pone.0061700 (PMC3630120; doi:10.1371/journal.pone.0061700)

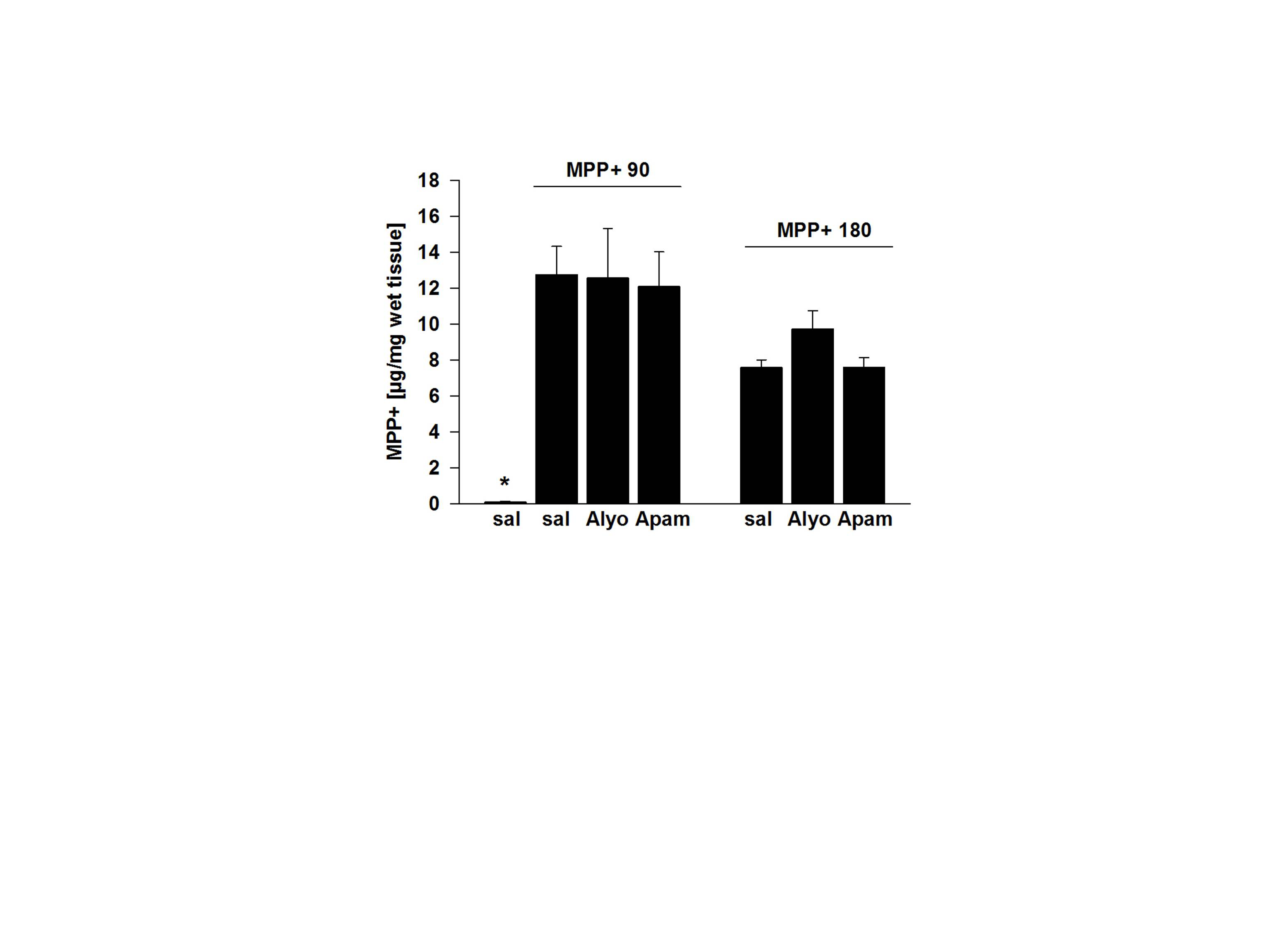

Supplement: Figure S1 — Effects of bee venom (Alyo) and apamin (Apam) on striatal MPP+ levels. The figure shows striatal MPP+ levels at 90 and 150 minutes after MPTP/probenecid treatment and 36 hours after bee venom, apamin or saline treatment, respectively. At no time point MPP+ levels were altered by the treatment. (TIF) [file pone.0061700.s001.tif]

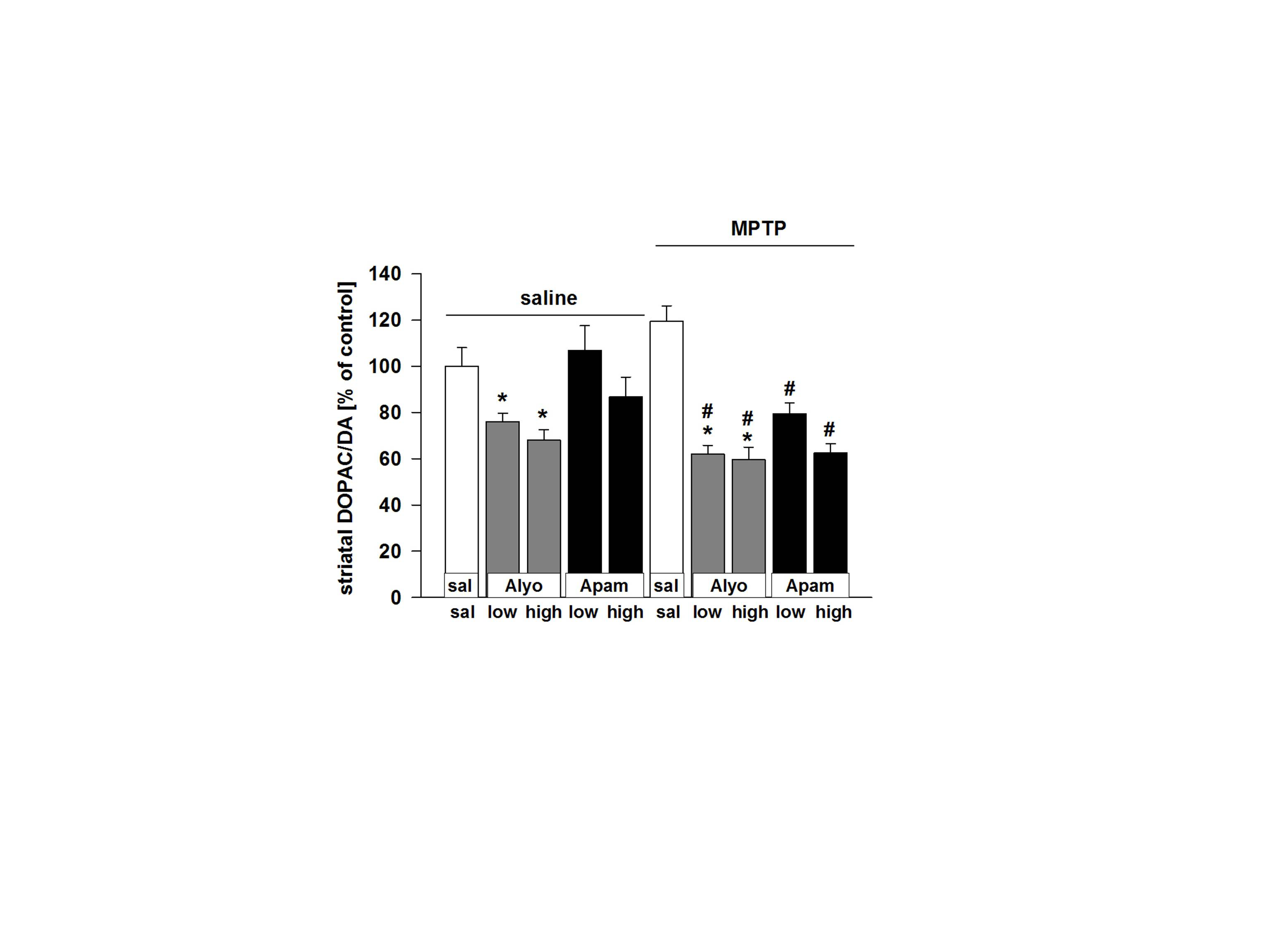

Supplement: Figure S2 — Striatal DOPAC/DA ratios in MPTP/probenecid-treated mice treated with bee venom (Alyo), apamin (Apam) or saline (Sal). Data are expressed as percentage of saline-injected mice. Actual values for saline/saline treated animals DOPAC/DA ratio were 0.59±0.05. Data represent mean values ± s.e.m of 5–8 animals. *p<0.05 compared to control values, #p<0.05 for pairwise comparison. (TIF) [file pone.0061700.s002.tif]

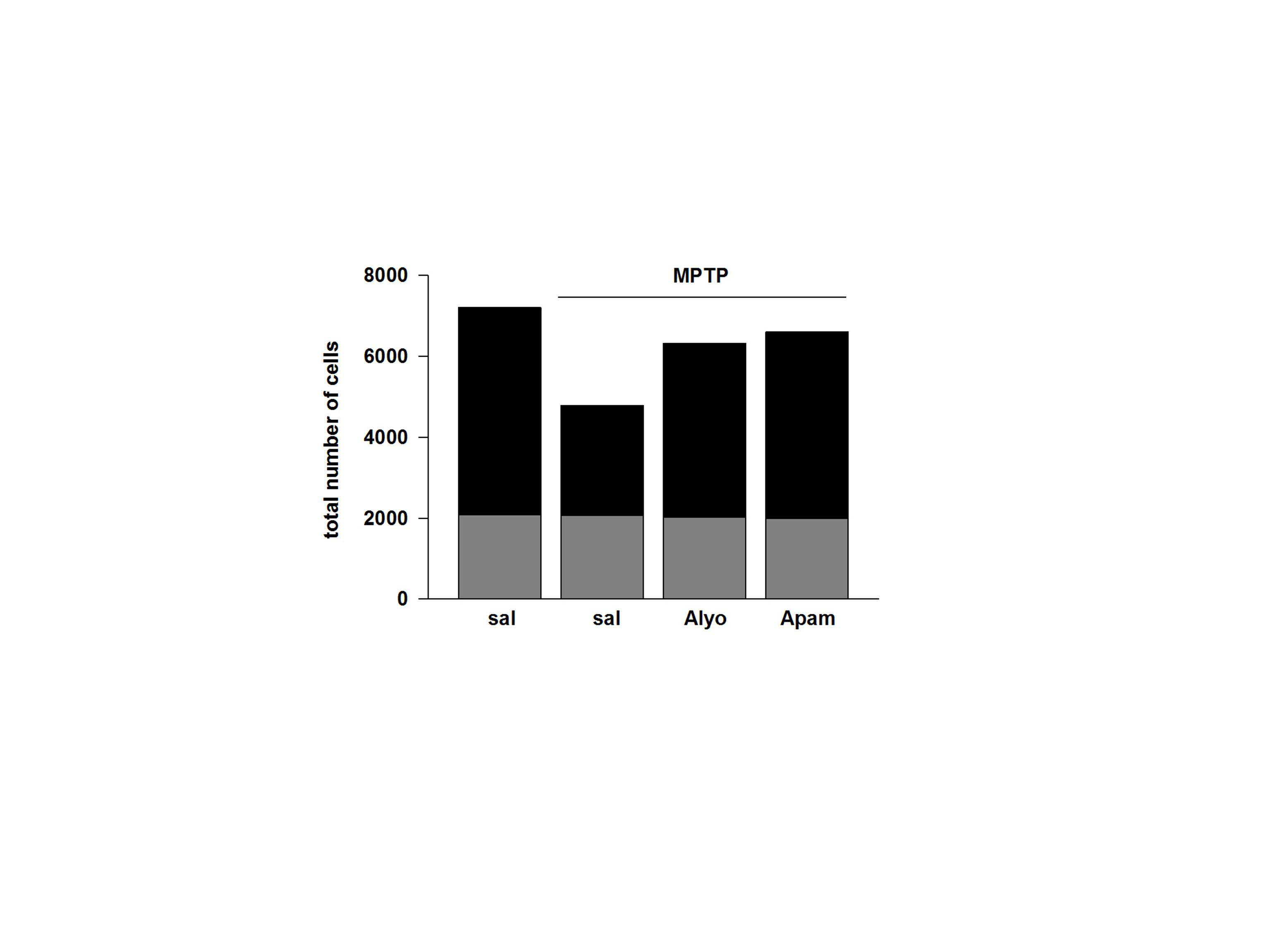

Supplement: Figure S3 — Total number of neurons in the SNpc. The graph represents the absolute number of TH+ (black) and TH− (grey) neurons in the SNpc of mice treated with saline/saline, MPTP/saline, MPTP/high Alyostal, and MPTP/high Apamin. Data represent mean of 5–8 animals per group. (TIF) [file pone.0061700.s003.tif]
