# Supplementary material for: Bee Venom and Its Component Apamin as Neuroprotective Agents in a Parkinson Disease Mouse Model
Source: PLoS One. 2013 Apr 18;8(4):e61700. doi: 10.1371/journal.pone.0061700 (PMC3630120; doi:10.1371/journal.pone.0061700)
Supplement: Supporting Information S1 — In the supporting information section technical details of MPP+ analysis after pre-treatment with either bee venom or apamin are specified. (DOC) [file pone.0061700.s004.doc]

**Supporting information**

**Striatal MPP+ levels after pre-treatment with bee venom and apamin**

To exclude that MPP+ levels were altered following apamin or bee venom treatment, we treated animals with high dosage of these compounds or saline, respectively 1 day prior to MPTP injection according to the paradigm used in the study (Fig 1). 90 and 150 minutes after MPTP/probenecid (25mg and 250 mg/kg) injection mice were killed (n=5 per group) and striata were placed into 250 µl perchloric acid. Eluates were delivered at a rate of 1.0 mL/min via a mobile phase consisting of 2.7 g potassium dihydrogen sulphate dissolved in 697 mL acetonitrile per liter (adjusted to pH 2.5 with H_3_PO_4_) onto a reversed phase C18 column (250 x 4 mm, pre-column 5 x 4 mm) filled with Nucleosil 100 C18 (Knauer, Berlin, Germany). The sample injection volume was 10 µL. MPP+ was assessed by UV detection at a wavelength of 295 nm. At no time point treatment altered striatal MPP+ levels (see Fig S1).
