# Supplementary material for: Bee Venom and Its Component Apamin as Neuroprotective Agents in a Parkinson Disease Mouse Model
Source: PLoS One. 2013 Apr 18;8(4):e61700. doi: 10.1371/journal.pone.0061700 (PMC3630120; doi:10.1371/journal.pone.0061700)
Supplement: Table S1 — Statistical details. The table gives all statistical details as dependent variable, the performed test (all ANOVAs were followed by a post-hoc Holm-Sidak test for all pairwise comparisons), the degrees of freedom (dF), F, and p-values. (DOCX) [file pone.0061700.s005.docx]

| **Dependent variable** | **test** |  | **dF** | **F** | **P** |
| --- | --- | --- | --- | --- | --- |
| Cells | One-way ANOVA | | 9 | 10,664 | <0,001 |
| DA | One-way ANOVA | | 9 | 31,598 | <0,001 |
| HVA/DA | One-way ANOVA | | 9 | 28,990 | <0,001 |
| DOPAC/DA | One-way ANOVA | | 9 | 11,953 | <0,001 |
| MPP+ | One-way ANOVA | | 6 | 8,679 | <0,001 |
| Ilβ1 | One-way ANOVA | | 5 | 0,290 | 0,915 |
| IL6 | One-way ANOVA | | 5 | 5,701 | <0,001 |
| TNFα | One-way ANOVA | | 5 | 6,335 | <0,001 |
| NOF | Two-way ANOVA with repeated measurements | Treatment | 5 | 1,566 | 0,193 |
|  |  | Run | 1 | 11,465 | 0,002 |
|  |  | Treatm x run | 5 | 5,086 | 0,001 |
| RotaRod | Two-way ANOVA with repeated measurements | Treatment | 5 | 8,466 | <0,001 |
|  |  | Run | 2 | 25,865 | <0,001 |
|  |  | Treatm x run | 10 | 3,173 | <0,001 |
| Cx I /CS activity | Two-way ANOVA | Treatment | 2 | 0,172 | 0,843 |
|  |  | MPTP | 1 | 16,305 | 0,001 |
|  |  | Treatm x MPTP | 2 | 0,0589 | 0,943 |
